# Burnout among Nurses and Doctors Working at a Tertiary Care Government Hospital: A Descriptive Cross-sectional Study

**DOI:** 10.31729/jnma.8577

**Published:** 2024-05-31

**Authors:** Sunil Kumar Shah, Richa Sinha, Pratik Neupane, Gobinda Kandel

**Affiliations:** 1 Department of Psychiatry, Bharatpur Hospital, Chitwan, Nepal; 2 Department of Internal Medicine, Bharatpur Hospital, Chitwan, Nepal

**Keywords:** *burnout*, *doctors*, *government*, *nurses*

## Abstract

**Introduction:**

Work environment related feelings of dissatisfaction, exhaustion, decreased interest and isolation is common. Burnout among health professionals has been on rise at every stage of professional growth affecting wellness of service providers, patient care and health care organizational efficiency. Assessment of burnout among health care workers from government setup in the current context in this post COVID era in our socio-geographical context has become essential. The aim of this study was to find the prevalence of burnout among nurses and doctors working at a tertiary care government hospital in Nepal.

**Methods:**

This descriptive cross sectional study was conducted among nurses and doctors working at a tertiary level government hospital from 10^th^ May 2022 to 9^th^ Nov 2022 after approval from Institutional Review Committee of the same institute. Nurses and doctors available on duty, from all ages were included. Trainees and students, those unable to participate due to their illness, on leave, known cases of mental illness were excluded. The point estimate was calculated at 95% Confidence.

**Results:**

Among 180 participants, the prevalence of moderate burnout was 94 (52.22%) (44.92-59.51, 95% Confidence Interval). Among nurses 72 (50%), while in doctors 22 (61.11%) had moderate burnout. Out of those with moderate levels of burnout, the majority of 66 (52.80%) were in the age group 26-50 years.

**Conclusions:**

The prevalence of burnout among nurses and doctors is high, similar to other studies done in similar settings.

## INTRODUCTION

Initially defined by clinical psychologist Herbert, in 1974, burnout is a syndrome observed among people working in demanding services, characterized by exhaustion, emotional and psychosomatic difficulties associated with chronic job stress.^1^ Loss of energy, negative feelings related to job, and feeling ineffective are the three important dimensions of burnout.^2^

Burnout among nurses and doctors are highly prevalent warranting urgent attention and action globally affecting healthcare professionals' life, their families, society and patient care quality.^3,4^

Several studies have been conducted on burnout among healthcare providers in Nepal but here we have focused on burnout among nurses and doctors working at a tertiary level government hospital.

The aim of this study was to find prevalence of burnout among nurses and doctors working at a government tertiary care center.

## METHODS

This descriptive cross-sectional study was conducted at a government tertiary hospital. This study was conducted from 10^th^ May 2022 till 9^th^ Nov 2022. Nurses and doctors working at the facility were enrolled in the study after Institutional Review Committee approval (Reference number: 078/079-20) with informed consent. Only nurses and doctors currently employed at any department of the center were approached for enrollment. Those unable to participate due to their illness, on leave, known cases of mental illness, students and trainees were excluded. Convenient sampling method was adopted.


$$\begin{array}{ll} \text{n} & ={\text{Z}}^{2}\times \frac{\text{p}\times \text{q}}{{\text{e}}^{\text{2}}}\\ & =1.96^{2}\times \frac{0.9055\times 0.945}{0.05^{2}}\\ & =131.5\sim 132 \end{array}$$

Where,

n= minimum required sample size
Z= 1.96 at 95% Confidence Interval (CI)
p= 90.55, expected prevalence taken as % from previous study^5^
q= 1-p
e= margin of error, 5%

The minimum sample size was calculated to be 132 considering 5% margin of error and 95% confidence interval. However, out of 200 nurses and doctors approached, 180 completed and returned the proforma with a response rate of 90%.

Data collected with self-structured questionnaire for baseline characteristics and self-report questionnaire, Professional Quality of Life Scale Version 5 (ProQoL-5) Nepali version was checked for completeness and coded with serial number. The ProQOL-5 is a 30 item self-report measure on a 5-point scale from 1=never to 5=very often. Scores for 3 subscales are computed by aggregating the scores of 10 specific items. For calculating burnout score items 1, 4, 15,17 and 29 were reversed before calculating. To find scores on burnout questions 1,4, 8,10,15,17,19,21,26 and 29 were considered. Scores less than 22 was low level, between 23 and 41 was moderate and scores 42 or more was considered as high level of scale for burnout, secondary traumatic stress and compassion satisfaction.^6,7^

Data was entered in excel and analyzed using STATA 15.1. Descriptive statistics were computed. Categorical variables were expressed in frequency and percentage while, for continuous variable mean and standard deviation were calculated. Point estimate and 95% CI were calculated.

## RESULTS

Among the total 180 participants, prevalence of moderate burnout was 94 (52.22%) (44.92-59.51, 95% CI) (Table 1).

**Table 1:** Prevalence of burnout at work place (n = 180).

Level of burnout at work	n (%)	95% CI
Low	85 (47.22)	39.92-54.51
Moderate	94 (52.22)	44.92-59.51
High	1 (0.56)	0.53-1.65

Out of the 36 participant doctors, 22 (61.11%) (53.9968.23, 95% CI) had moderate burnout while among 144 nurses, 72 (50%) (46.27-53.73,95% CI) had moderate burnout (Table 2).

**Table 2:** Burnout among doctors and nurses (n= 180).

Level of burnout at work	n (%)	95% CI
**Among doctors**		
Low	14 (38.89)	31.77-46.01
Moderate	22 (61.11)	53.99-68.23
High	-	-
**Among nurses**		
Low	71 (49.31)	45.58-53.04
Moderate	72 (50)	46.27-53.73
High	1 (0.69)	0.23-1.69

Mean age of the participants was 31.44± 8.32 years. Participants were mainly from 26-50 years' age group, 125 (69.44%). Majority of them were females, 149 (82.78%). One hundred and twenty-three (68.33%) of the participants were married. Out of the total participants, 144 (80%) were nurses and 36 (20%) were doctors. Local residents were more in the participants, 137 (76.54%) (Table 3).

**Table 3:** Demographic data of participants (n= 180).

Variables	n (%)
**Age (years)**	
< 25	47 (26.11)
26 - 50	125 (69.44)
>50	8 (4.44)
**Gender**	
Female	149 (82.78)
Male	31 (17.22)
**Marital status**	
Married	123 (68.33)
Unmarried	57 (31.67)
**Religion**	
Hindu	165 (91.67)
Muslim	1 (0.56)
Buddhist	13 (7.22)
Christian	1 (0.56)
**Ethnicity**	
Brahmin	100 (55.56)
Chhetri	24 (13.33)
Gurung	10 (5.56)
Madhesi	2 (1.11)
Newar	18 (10)
Dalit	6 (3.33)
Magar	10 (5.56)
Tamang	10 (5.56)
**Local resident**	
Yes	137 (76.54)

By years of work experience, 89 (49.44%) had been working for one to five years. One hundred and nine (60.56%) of the study population has been working at the job position of staff nurse. As per stability of job, a majority of 128 (71.11%) were having temporary jobs while only 52 (28.89%) had permanent jobs. Most of them had no other major professional work besides a job at the government hospital, 156 (86.67%). Only 17 (9.44%) of them believed they would recommend their children to opt for the medical profession as a career. Majority had the responsibility of taking care of patients, and didn't have academic or administrative responsibilities, 149 (82.78%) (Table 4).

**Table 4:** Job related description of variable data (n= 180).

Categories	n (%)
**Job experience**	
1 to less than 5 years	89 (49.44)
10 completed years and above	33 (18.33)
5 years to less than 10	51 (28.33)
less than 1 year	7 (3.89)
**Job position**	
Auxiliary nurse midwife	14 (7.78)
Consultant doctor	17 (9.44)
Medical and dental officer	19 (10.56)
Nursing officer	21 (11.67)
Staff nurse	109 (60.56)
**Job appointment category**	
Permanent	52 (28.89)
Temporary	128 (71.11)
**Other additional job**	
No	156 (86.67)
Yes	24 (13.33)
Career of child as a medical professional	
Yes	17 (9.44)
**Job responsibilities**	
Academics	20 (11.11)
Administrative work	1 (0.56)
Taking care of patients	149 (82.78)
Taking care of patients, academics	5 (2.78)
Taking care of patients, academics, administrative work	3 (1.67)
Taking care of patients, administrative work	2 (1.11)

## DISCUSSION

The aim of this study was to find prevalence of burnout among nurses and doctors. Prevalence of moderate burnout was 94 (52.22%) (44.92-59.51, 95% CI). Among nurses, 71 (49.31%), 72 (50%) and 1(1.96%) had low, moderate and high levels of burnout. While in doctors, 14 (38.89%), 22 (61.11%) and 0 had low, moderate and high levels of burnout.

Several other studies have reported a similar level of burnout among healthcare providers. A study done in 2020-21 in Nepal, found total burnout score among health professionals ranged from low (9.5%), moderate (89.5%) to high (1%); while 120 (88.9%) doctors and 47 (92.2%) nurses were found having moderate level of burnout.^5^ In another 2020 study done among doctors in Kathmandu found that burnout level was low in 48 (27.60%), moderate in 126 (72.4%) and high in 0 (0%).^8^ Likewise a study done in nurses in Malaysia in 2019 found overall burnout among 24.4% (17.7-32.6, 95% CI).^9^ Similarly, a study from Norway done in 2021 during the COVID-19 pandemic reported high prevalence of burnout among health professionals.^10^

In an observational study conducted in Italy in 2020 during the pandemic, 752 (38.3%) of participants had high emotional exhaustion, 911 (46.5%) low professional efficacy and 519 (26.5%) high cynicism. Prevalence of burnout was high in health workers involved in care of covid cases, junior health workers and those already with psychosocial issues. Burnout phenomenon was highlighted to be of significant concern impacting quality of life of caregivers and quality of the care they would provide.^11^ Another 2021 mixed method study in pandemic from Jordan, the prevalence of burnout among physicians was 57.7%. Female sex, high workload, long working hours, working at night shifts, lack of protective equipment, infection etc. were found affecting burnout significantly.^12^ Further, a 2017 systematic review reported that burnout was seen moderate to high among both Arabic and Western countries health workers.^13^ Studies conducted during pandemic has found note worthily higher level of burnout among health workers. Burnout among health professionals has emerged as an epidemic with negative impact on health worker's health, patient care, and the whole health care delivery system.^14^ On the contrary to our study, a 2019 survey in the University of Kentucky, participants had low burnout, mean score. Nurses caring pediatric population and those without behavioral health problems had lesser burnout.^15^ Some studies report low level of burnout among healthcare professionals related to integrated health practice incorporating self-care, self-compassion, empathy, mindfullness.^16^

This study has been conducted among workers at a tertiary care government setup with those who experience the challenges and opportunities of serving at a multispecialty referral hospital. Doctors and Nurses having various kinds of duties and responsibilities could be included in the hospital. Due to restraints of resources, extending such study to healthcare providers among various other levels and locality could not be done. Factors contributing to burnout has not been looked into.

## CONCLUSIONS

Prevalence of burnout among nurses and doctors at this hospital was found high, similar to other studies. Burnout among nurses and doctors working in government setup warrants attention. Considering the prevalence of burnout recommendation to look into this area for policy makers seems advisable.
